# Neighboring plants divergently modulate effects of loss-of-function in maize mycorrhizal phosphate uptake on host physiology and root fungal microbiota

**DOI:** 10.1371/journal.pone.0232633

**Published:** 2020-06-17

**Authors:** Izabela Fabiańska, Lina Pesch, Eva Koebke, Nina Gerlach, Marcel Bucher

**Affiliations:** 1 Institute for Plant Sciences, Cologne Biocenter, University of Cologne, Cologne, Germany; 2 Cluster of Excellence on Plant Sciences, University of Cologne, Cologne, Germany; University of California Berkeley, UNITED STATES

## Abstract

Maize, a main crop worldwide, establishes a mutualistic symbiosis with arbuscular mycorrhizal (AM) fungi providing nutrients to the roots from soil volumes which are normally not in reach of the non-colonized root. The mycorrhizal phosphate uptake pathway (MPU) spans from extraradical hyphae to root cortex cells housing fungal arbuscules and promotes the supply of phosphate to the mycorrhizal host in exchange for photosynthetic carbon. This symbiotic association with the mycobiont has been shown to affect plant host nutritional status and growth performance. However, whether and how the MPU affects the root microbial community associated with mycorrhizal hosts in association with neighboring plants, remains to be demonstrated. Here the maize germinal Mu transposon insertion mutant *pht1;6*, defective in mycorrhiza-specific Pi transporter *PHT1;6* gene, and wild type B73 (wt) plants were grown in mono- and mixed culture and examined under greenhouse and field conditions. Disruption of the MPU in *pht1;6* resulted in strongly diminished growth performance, in reduced P allocation to photosynthetic source leaves, and in imbalances in leaf elemental composition beyond P. At the microbial community level a loss of MPU activity had a minor effect on the root-associated fungal microbiome which was almost fully restricted to AM fungi of the Glomeromycotina. Moreover, while wt grew better in presence of *pht1;6*, *pht1;6* accumulated little biomass irrespective of whether it was grown in mono- or mixed culture and despite of an enhanced fungal colonization of its roots in co-culture with wt. This suggested that a functional MPU is prerequisite to maintain maize growth and that neighboring plants competed for AM fungal Pi in low P soil. Thus future strategies towards improving yield in maize populations on soils with low inputs of P fertilizer could be realized by enhancing MPU at the individual plant level while leaving the root-associated fungal community largely unaffected.

## Introduction

Phosphorus (P) is one of the macronutrients essential for plant growth and productivity, however, on a global scale it is a slowly disappearing nutrient and unlike some finite resources like oil, for which alternatives can be found, there are currently no substitutes for P fertilizers [1,2]. Plants can take up P as inorganic orthophosphate (Pi, phosphate), a form which is often unavailable in soils due to high fixation to soil which results in a slow diffusion rate [3]. Most land plants, including important crops like maize, form arbuscular mycorrhizal (AM) symbiosis with soil based fungi from the subphylum Glomeromycotina [4,5] in response to Pi limitation. This biotic association generally enhances the capacity of plants to acquire nutrients and water from the mycorrhizosphere which expands the soil volume that can be mined for nutrients far beyond the rhizosphere through the development of an underground hyphal network formed by AM fungi [6,7]. In the root cortical cells of host plants AM fungi form arbuscules, highly branched hyphal structures, which are surrounded by the plant plasma membrane constituting the periarbuscular membrane (PAM) [8]. In these root cells, trading for nutrients between plant host and AM fungi takes place; fungi deliver essential nutrients to the plant (mainly Pi) in exchange for carbon in the form of sugars and fatty acids provided by the photobiont [9–12]. AM host plants possess specific Pi transporters from the PHT1 family targeted to the PAM, which actively transport Pi delivered by the fungus to host cells [13–15]. This pathway of soil-derived mineral P uptake is known as the mycorrhizal Pi uptake (MPU) pathway, which can operate exclusively or in parallel to direct Pi uptake (DPU) from the soil solution across the root epidermis [16]. Subsequently Pi transverses from the cortex through the endodermis into the stele and from there to aboveground organs. Interestingly, the Pi delivered via MPU can highly outweigh the amount of nutrient delivered by DPU [17], thus highlighting the importance of mutualistic AM symbiosis in terrestrial ecosystems. The expression of plant Pi transporter genes involved in MPU is locally and cell -autonomously controlled during AM fungal colonization of root cortical cells [18–20] and is tightly regulated by transcription factors which bind to distinct *cis*-regulatory elements present in respective gene promoters [21–23].

The maize genome contains 13 putative genes encoding PHT1 transporters [24,25], from which four were specifically induced at transcript level in the cells colonized by AM fungi under low Pi conditions relative to non-colonized controls [25,26]. The largest increase in transcript levels during AM symbiosis was observed for *Pht1;6* [24–27], implying its involvement in Pi transport via the MPU. Moreover concomitant with Pht1 gene expression extraradical hyphal network density correlates with Pi uptake efficiency in maize [25]. Indeed, impaired development of functional AM symbiosis was evident in the *Pht1;6* loss-of-function mutant *pht1;6* (designated “mu” hereafter) which was associated with the accumulation of small, septated arbuscules and strongly reduced uptake of Pi via the MPU in sand-soil mixture inoculated with *Rhizophagus irregularis* [26].

The molecular components underlying MPU activity and its contribution to host plant performance were mainly deduced from reductionist experiments based on binary interactions with a limited spectrum of model AM fungi which were conducted under laboratory conditions [13,28]. In nature, plants interact with a plethora of microorganisms including among many others a diversity of AM fungi. Therefore a conceptual framework is required illustrating that interactions among mycorrhizal plants and their associated microbiota are critical for the establishment and the maintenance of host-microbial homeostasis and plant performance [29]. In this context the holobiont concept has emerged as a theoretical and experimental framework to study the interactions between plant hosts and their associated microbial communities in various types of ecosystems. The holobiont concept has been developed by Margulis for the intimately integrated (and usually obligate symbiotic) associations between insects and their bacterial endosymbionts, whereas the insect holobiont was regarded as an ecological unit [30,31]. In natural environments, however, plants interact with neighboring plants and it is therefore worth considering whether in a population each plant represents a unit with its microbial community (a holobiont per definition) or whether plant-plant interactions impact root microbiomes. The latter would require revisiting Margulis´ definition of plants as holobionts in their natural environment without being incomplete.

In field experiments performed in 2006, 2007, and 2009 in low-P soil *pht1;6* grew much less and exhibited strongly reduced cob number, highlighting the importance of the MPU for agricultural maize production at low P fertilization [26]. Notably, the reduced-colonization phenotype of *pht1;6* could partially be rescued by growing mutant plants together with well colonized B73 wild type (so called nurse plants) [26]. This trans-complementation of mutants defective in MPU is likely accomplished through carbon resources provided to the AM fungus by the nurse plant [26,32,33].

Thanks to the development of high-throughput sequencing technologies, the knowledge on ecosystem functioning has advanced in recent years by studies including host-associated microbial communities [34–36]. Variation in maize host microbiota was shown to depend on different factors like the biogeography of the field, the plant genotype, host root exudation, and application of biocontrol agents [37–40]. Importantly, the fungal community in maize roots was shown to be shaped by soil P content and root type [41]. However, how maize mycorrhizal microbial community homeostasis is affected by functional AM symbiosis traits is elusive. Recent studies performed with mycorrhizal *Lotus japonicus* mutants disrupted in the common symbiosis signaling pathway (CSSP) or downstream processes showed that an almost complete depletion of Glomeromycotina taxa and impaired arbuscule formation in roots were accompanied by an enrichment of few Ascomycota fungi and shifts in bacterial communities under greenhouse conditions [42,43]. On the other hand, knocking out CSSP genes didn´t affect the life cycle of the growth-promoting basidiomycete fungus *Piriformospora indica* (also known as *Serendipita indica*) in *L*. *japonicus* nor did silencing of CCaMK affect bacterial or fungal communities in roots of field-grown *Nicotiana attenuata* [44,45]. The results with tobacco suggested that non-legume plants impose less impact on their root-associated microbiota relative to legumes which are capable of establishing symbioses with AM fungi and with nitrogen-fixing rhizobial bacteria [45]. Alternatively the host-mediated effect on the microbiota could be accelerated in pot-grown plants, but became insignificant under field conditions with stochastic environmental variation and seasonal fluctuations in resource availability [46]. Additionally, phylogenomic studies of mycorrhiza-specific Pi transporter genes suggested that recruitment of Pht1 genes appeared late during the evolution of the AM symbiosis [47] and therefore the MPU might not greatly affect root colonization by AM and other fungi.

Here, we examined a loss-of-function maize mutant defective in the mycorrhiza-dependent Pi transporter PHT1;6 under field and controlled greenhouse conditions to test the hypothesis that plant growth performance and interdependencies with fungal microbiome diversity and the plant nutritional status are affected by the MPU pathway of the host and its plant neighbor. The results will support a pivotal role of MPU in shaping a beneficial microbiome which promotes crop productivity and highlight mycorrhizal plant-plant interactions as a fundamental factor in the mutualistic AM symbiosis.

## Materials and methods

### Plant growth conditions

*Zea mays* (maize) inbred line B73 (wt) and mutant *pht1;6* (mu) in B73 background (5th backcross generation), carrying the Mu transposable element insertion in *Pht1;6* gene [26] were used in this study. Two experiments were performed, first under greenhouse, second under field conditions.

In the greenhouse experiment which was performed in 2014 (GH 2014), maize seeds were surface sterilized (soaked in 70% ethanol for 2 min, then in 23% sodium hypochlorite, washed with dd H_2_O) and sown in approx. 15 cm distance into +[NPK] soil harvested from agricultural field at Agroscope Reckenholz, Switzerland in Autumn 2013 (1, soil characteristics published in 2). Two maize plants were grown in one 7 L pot filled with 5.6 kg of soil, in the following combinations: two wild type plants in one pot (wt_wt pot), wild type plant with *pht1;6* mutant plant in one pot (wt_mu), and two *pht1;6* mutant plants in one pot (mu_mu, S1 Fig), in the greenhouse (24/20°C day/night temperature) for 9 weeks. The pots were regularly randomized and watered with dH_2_O to maintain the soil humidity at approx. 75% of its water-holding capacity. The plants were sampled separately. One fully developed leaf (source leaf) from each plant was collected for elemental analysis with ICP-MS. The maize shoot was dried in an oven at 60°C and dry weight was determined. The roots were separated and used to scoring fungal colonization and for microbial community analyses by ARISA and Illumina sequencing.

The second experiment was performed in 2015 (Field 2015) in agricultural fields at Agroscope Reckenholz in Switzerland [26]. B73 and *pht1;6* seeds were sown in the alternative order at the south field border of +[NPK] (fully fertilized), -[P] +[NK] (not fertilized with P since 1989) and–[NPK] (not fertilized with N, P and K since 1989) fields [26] (S1 Fig) with approx. 30 cm distance between the plants. Next to this border line, a line including just B73 plants was grown to separate the experimental plants from the hybrid maize grown on the remaining field. Plants were harvested in August after 3 months growth. At this point the plants flowered and started to generate cobs, but have been vital. The plants were sampled similarly as in GH 2014 experiment.

### Multi-elemental composition analysis using ICP-MS

For ICP-MS measurements, dried source leaves were cut with a ceramic scissor into small pieces to homogenize the material. 100mg of the source leaf material was then digested in 5 ml of concentrated HNO_3_ for around 2h at 100° C. Afterwards, the solutions were filtered (Whatman® filter grade 1, GE Healthcare Life Sciences, USA) to exclude solid particles. Determination of 22 elements was performed with an Agilent 7700 ICP‐MS (Agilent, http://www.home.agilent.com) following the manufacturer's instructions.

### Root and rhizosphere sampling and DNA extraction

Rhizosphere and root samples were collected using a fractionation method described previously [48], with additional 20 min washing in PBS buffer after sonication of root sample. The root samples were grinded before DNA extraction in liquid nitrogen using ceramic mortar and pestle. Subsequently, total genomic DNA from root and rhizosphere samples was extracted with FastDNA Spin Kit for Soil (MP Biomedicals, Solon, USA) according to the manufacturers’ instructions. Number of samples collected in the greenhouse and field experiments is indicated in S1 and S2 Tables, respectively.

### Automated Ribosomal Intergenic Spacer Analysis (ARISA)

Amplification of the ITS region amplification was performed using the ITS1-F *FAM and ITS4R primer pair for fungi [49] in a Verity Thermal Cycler (Applied Biosystems, Carlsbad, CA, USA) in 50 μL reactions containing 10 ng of DNA template, 0.5 μM of each primer, 400 μM of each nucleotide, 1.25 mM MgCl2, 0.05 U μL^-1^ of G2 Flexi DNA Polymerase (Promega, Mannheim, Germany) and 1X G2 Flexi DNA Polymerase buffer. PCR products were diluted with sterile ddH2O (1:100). The capillary electrophoresis for fragment analysis was performed at the Cologne Center of Genomics (CCG, Cologne, Germany) using GeneScan ROX1000 Size Standard (ThermoFisher Scientific, USA) as the internal length standard. Electropherograms were analyzed with Peakscanner v1.0 (Life Technologies, USA) and T-Rex software [50] (differentiation of peaks by 2 bp, analysis of peak heights).

### ITS2 amplicon sequencing

ITS2 amplicons were generated using a two-step PCR method using ITS9 and ITS4 primers, as described before [48,51]. The obtained paired-end reads were processed in Mothur version 1.37.3 using a custom pipeline [48] and the UNITE fungal ITS database (Version 7.2, release 1.12.2017) [52,53]. The raw ITS2 sequencing data is deposited at NCBI sequence read archive under Bioproject PRJNA548861.

### Statistical analyses

R studio (version 3.2.1) was used for statistical analyses [54]. The operational taxonomic unit (OTU) table was used to quantify OTU relative abundances which were Log10 (X+1) transformed. This final transformed OTU table was used to calculate Bray-Curtis dissimilarities between samples using the ‘vegdist’ function of the vegan package [55]. The ‘dudi.pco’ and ‘s.class’ functions from the ade4 package [56] were used to conduct the principal coordinates analyses (PCoA). Permutational multivariate analysis of variance (PerMANOVA) on Bray-Curtis dissimilarities was conducted using the ‘adonis’ function of the vegan package (at *P* < 0.05, 10,000 permutations). OTUs showing differences in their relative abundance between mutant (*pht1*.*6*) and wt roots were identified using the SIMPER function from vegan package. The average relative abundances of these OTUs were used to generate a heatmap using hierarchical clustering (with one minus Pearson’s correlation and average linkage). Relative abundances of the identified fungal OTUs in wt and mutant plants were further compared with Wilcoxon test (*P* < 0.05, FDR corrected). Unless otherwise stated means were compared using one-way ANOVA followed by Tukey’s HSD test (*P* < 0.05). Before that, the equality of variances for experimental groups of samples was tested with Levene's test. Correlation between leaf nutrient content and fungal order abundances was performed using Pearson’s method (|correlation coefficient| > 0.6, *P* < 0.05 after FDR correction).

### Root staining and microscopy

The colonization degree was assessed via trypan blue staining of roots harvested in 70% EtOH. The analysis was based on a magnified intersection method [57]. 10–15 root fragments of about 1cm length were placed onto microscopic slides. One hundred views (at 20 x magnification) were observed and classified into categories: hyphae, hyphae and arbuscule, hyphae and arbuscule and vesicle. Moreover, the percentage of total colonization was determined by summing up all colonization categories and dividing by total number of views observed.

## Results

### Biomass and elemental composition of wild type maize and *pht1;6* mutant in natural soil

**Fig 1:**
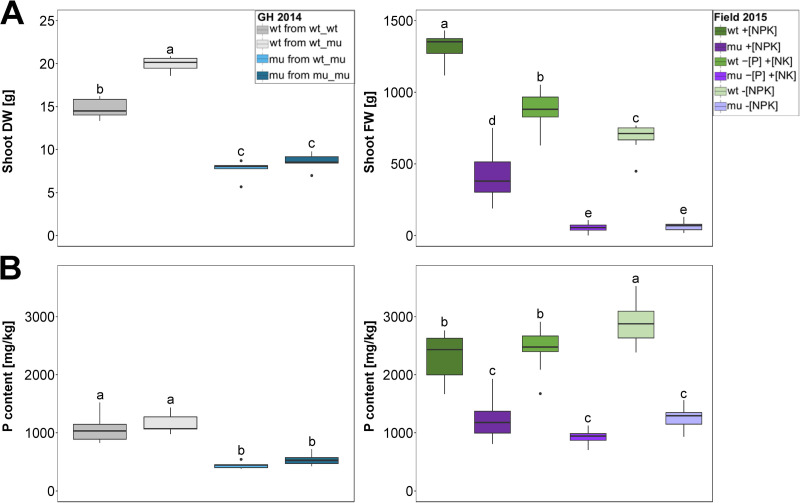
Physiological parameters of maize plants. A) Shoot dry weight (DW) in GH 2014 experiment and shoot fresh weight (FW) in Field 2015 experiment. Different letters indicate significant differences between the treatments. B) Concentration of phosphorus (P) in source leaves of *pht1;6* and wt plants in GH 2014 and in Field 2015 experiments. Different letters indicate significant differences between the treatments (ANOVA followed by Tukey’s HSD test, *P* < 0.05, n = 5 in GH 2014 or n = 8–10 in Field 2015 experiment).

To study effects of Pi uptake and neighboring plants on mycorrhizal physiology and microbiota assemblages, wt and *pht1;6* mutant (mu) plants were grown in pots in the greenhouse experiment (GH 2014) and under field conditions (Field 2015) (S1A and S1B Fig). In the GH 2014 experiment, *pht1;6* plants grew significantly smaller than wt, irrespective of the genotype of the neighboring plant (Fig 1A). Similarly, under field conditions *pht1;6* showed reduced above-ground (shoot) biomass production in all soils (Fig 1A). Interestingly, in the GH 2014 experiment, wt plants grown in the wt_wt configuration were smaller compared to wt grown in wt_mu culture (Fig 1A), suggesting more efficient nutrient uptake in wt relative to mu plants. In direct correlation with above results on biomass, *pht1;6* plants exhibited a reduced P content in source leaves compared to wt in all tested soils (Fig 1B), with “content” defined as amount of any type per mass of liquid or gas or solid system [58]. Moreover, in the field P limited maize growth in −[P] +[NK] and −[NPK] relative to +[NPK] soil, in both genotypes (Fig 1B). This indicated a major positive effect of MPU on plant growth and P content, independent of soil nutrient status.

Analysis of elemental profiles in source leaves revealed a reduction in elemental content in wt and mu maize grown in +[NPK] soil in the greenhouse experiment compared with the same genotypes grown in +[NPK] field soil (Student T-test *P* < 0.05, Figs 1B and S2 and S8 Table). Overall, elemental contents in wt and mu grown in the field reflected two distinct patterns with generally lower contents of P, S, and Cu (P-type) as opposed to a somewhat inverse pattern with higher contents of K, Mn, and Fe (K-type) in *pht1;6* relative to wt plants, especially in −[P] +[NK]) and −[NPK] soil low in P (S2 Fig). Mutants from mu_mu pots and mutants grown in −[P] +[NK] and −[NPK] soil accumulated significantly more Mn in source leaves compared with other plants. Wt plants grown in wt_wt pots in the greenhouse and wt grown in −[P] +[NK] field soil accumulated less Fe and Cu relative to mu from mu_mu pots or grown in the same field. In summary, the results on elemental content in source leaves indicated that the disruption of mycorrhiza-specific Pi transport had a pronounced negative impact on plant growth, leave P content and nutrient uptake from the soil substrate, and that this impact was stronger than the effect of soil P availability.

### Impact of MPU pathway on fungal structures in mycorrhizal roots

**Fig 2:**
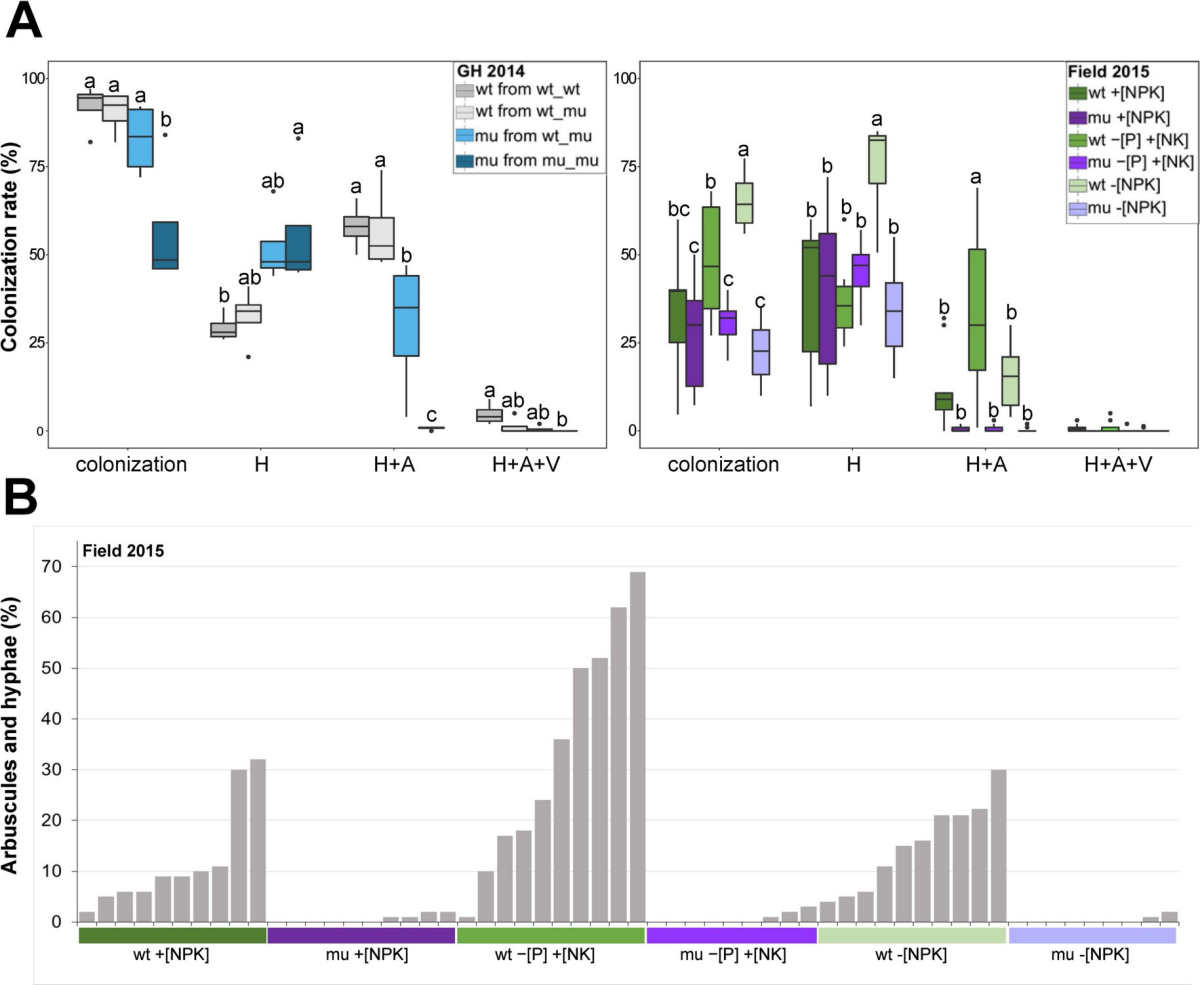
Fungal colonization degree in the roots of wt and *pht1;6* (mu) plants growing in an agricultural soil (+[NPK]) in the greenhouse and under the +[NPK],–[P] +[NK] and–[NPK] soil management type under field conditions. A) Percentage of plant roots with observable fungal hyphae (H) or hyphae with well-developed arbuscules (H+A) or hyphae with well-developed arbuscules and vesicles (H+A+V). ‘Colonization’ indicates the sum of ‘H’ plus ‘A’ plus ‘V’ representing the overall percentage of plant roots colonized by fungal structures (n = 5). B) Percentage of plant roots with hyphae with well-developed arbuscules in plants sampled in Field 2015 experiment. Different letters indicate significant differences between the treatments (ANOVA followed by Tukey’s HSD test, *P* < 0.05, applied on each category of fungal structures separately, n = 5 or 8–10, in GH 2014 and Field 2015 experiments, respectively).

Next we explored fungal morphology in mycorrhizal roots and quantified the percentage of root colonization and of mycorrhizal structures in trypan blue stained root samples collected in the two experiments from both maize genotypes. In GH 2014 plants grown in the mu_mu configuration showed a significantly lower degree of fungal colonization, mainly driven by a reduced abundance of AM fungi, shown by a strong reduction in arbuscule density relative to wt (Fig 2A). The mu plants grown with wt in wt_mu pots exhibited increased arbuscule formation compared with mu plants in monoculture, indicating that AM fungi could colonize mu roots in the presence of wt nurse plants. In the Field 2015 experiment, wt plants contained arbuscules, however at a lower degree compared with the pot experiment, whereas mu plants showed reduced but still detectable AM fungal colonization compared with wt in all soils, manifested by absence of arbuscules in the majority of mu roots. Six out of 10 samples tested in +[NPK], six out of nine in −[P] +[NK], and seven out of nine in −[NPK]. In contrast, all roots contained arbuscules in the wt (Fig 2B). This suggested that mu is strongly restricted in AM symbiosis formation under field conditions due to defective MPU. In summary, mu plants in comparison to wt exhibited a reduction in root AM fungal colonization in absence of nurse plants.

### Impact of *Pht1;6* knock-out on root-associated fungal community profiles

To study the impact of a disrupted MPU pathway on root-associated fungal microbiota in the GH 2014 experiment we used the intragenomic diversity fingerprinting method ARISA, which showed that the fungal community structure was primarily affected by the inspected compartment (i.e. root and rhizosphere) which became evident in the principal component analysis (PCoA, S3 Fig), confirmed by permutational multivariate analysis of variance (PERMANOVA on Bray–Curtis dissimilarities; *P* = 10^−4^, 40% of variance). The effect of the plants’ configuration in pots (i.e. plants from wt_wt and mu_mu, wt from wt_mu, mu from wt_mu) contributed overall 8% to the explained variance in fungal communities (PERMANOVA *P* = 0.002). In detail, a significant effect of the plants’ configuration in the pot on the fungal community structure could only be observed in the root compartment, where it accounted to 25% (PERMANOVA *P* = 2 x 10^−4^) of explained variability, whereas the rhizosphere compartment remained unaffected (PERMANOVA *P* = 0.061, S3 Fig). This implied that the plant genotype affected the microbiota inhabiting the root interior. Indeed, ARISA revealed that three fungal phylotypes out of overall 141 detected in roots (2.13%) differed in their abundance in wt plants grown in wt_wt pots versus mutants grown in mu_mu pots (Wilcoxon *P* < 0.05, FDR corrected). In the rhizosphere compartment none of the fungal phylotypes differed in relative abundance in mu relative to wt when plant genotypes were grown in monoculture. This suggested a minor contribution of the MPU pathway to maize root microbiota formation.

### Impact of *Pht1;6* knock-out on root-associated fungal taxa

**Fig 3:**
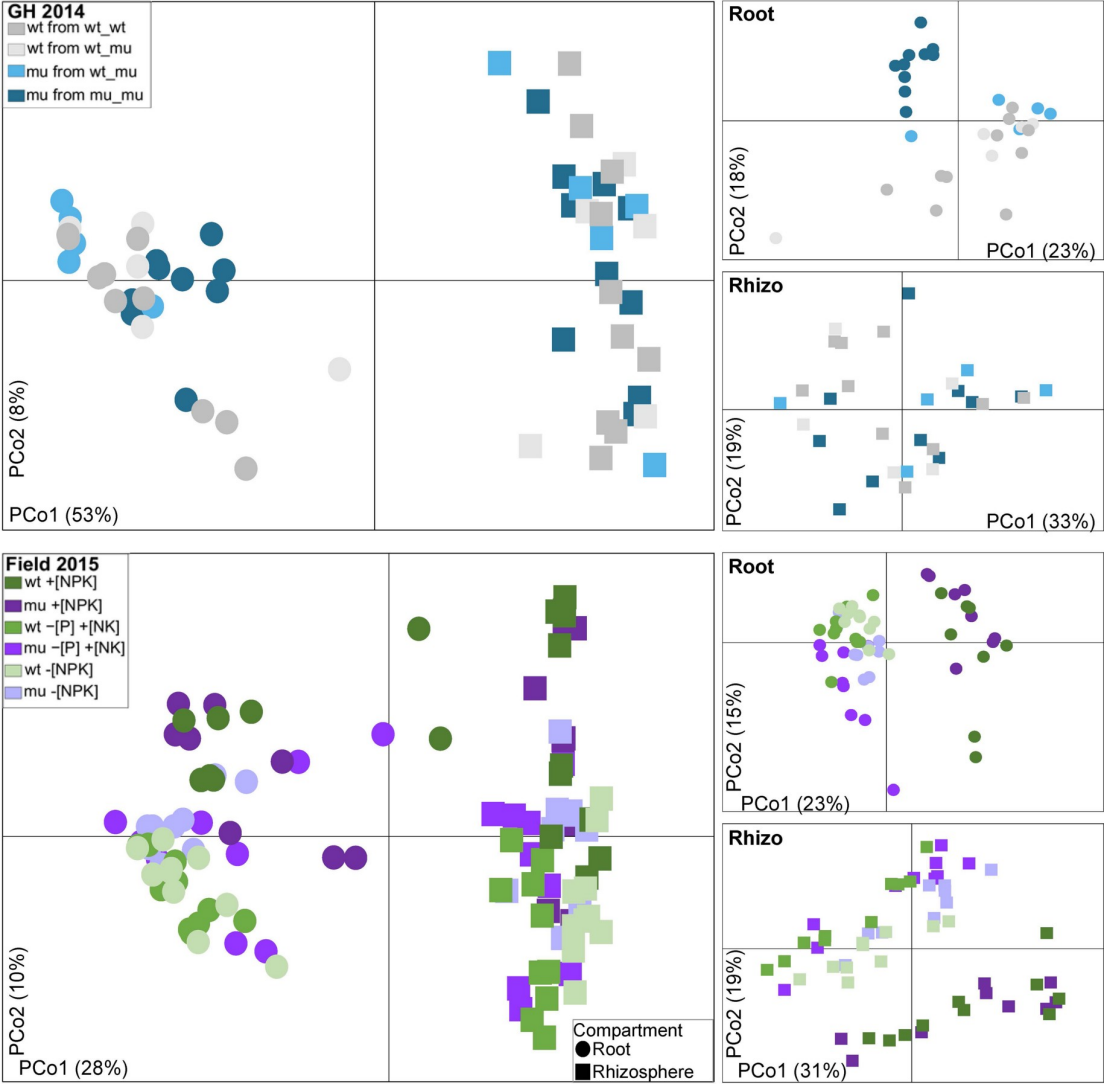
Comparison of fungal communities colonizing roots and rhizosphere of B73 wild-type (wt), and *pht1;6* plants (mu) visualized by principal coordinates analysis (PCoA) on Bray-Curtis dissimilarities of fungal communities in root and rhizosphere samples in GH 2014 experiment performed in +[NPK] soil and in field 2015 performed under the +[NPK],–[P] +[NK] and–[NPK] soil management types. Samples were colored according to the pot design.

To characterize the fungal members of root-associated microbiota we used Illumina-based amplicon sequencing targeting the ITS2 fragment in root and rhizosphere samples of mu and wt collected in the GH 2014 and Field 2015 experiments. On average, 38.936 high-quality fungal reads were obtained per sample, which were clustered at the 97% sequence similarity threshold, yielding 602 and 256 fungal OTUs in rhizosphere and root samples, respectively (S3 Table). Fungal alpha diversity estimated by Shannon’s H index was lower in the root compared to rhizosphere samples in both experiments, in all tested soils (Wilcoxon test *P* < 0.05, S4 Fig), implying a plant-mediated selection of soil fungi colonizing roots. There was no difference in fungal alpha diversity between mu and wt plants in both root and rhizosphere compartments in the greenhouse and field experiment which demonstrated that disruption of the MPU pathway left the total number of root-associated fungal taxa unaffected (S4 Fig). The overall fungal community structure (beta diversity) across two experiments was primarily affected by the plant compartment (PERMANOVA *P* = 10^−5^, 27% of variance) and the experiment (PERMANOVA *P* = 10^−5^, 21% of variance), which became evident in the PCoA on Bray–Curtis dissimilarities where the samples were separated according to the compartment type along the first principal component (PCo, 28% of variance) and according to the experiments along the second PCo (21% of variance) (S5 Fig and S4 Table). Overall, microbiome community structure at the fungal order level clearly differed between root and rhizosphere compartments, in that fungi belonging to the orders Glomerales, Paraglomerales, Xylariales, unclassified Basidiomycota, Pleosporales, Myrmecridiales, unclassified Sordariomycetes, Helotiales, Magnaporthales and Cantharellales were enriched in the root relative to the rhizosphere samples in both experiments (Fig 3, Wilcoxon *P* < 0.05), suggesting selection of particular soil-based fungal groups by maize plants for accommodation in their roots.

**Fig 4:**
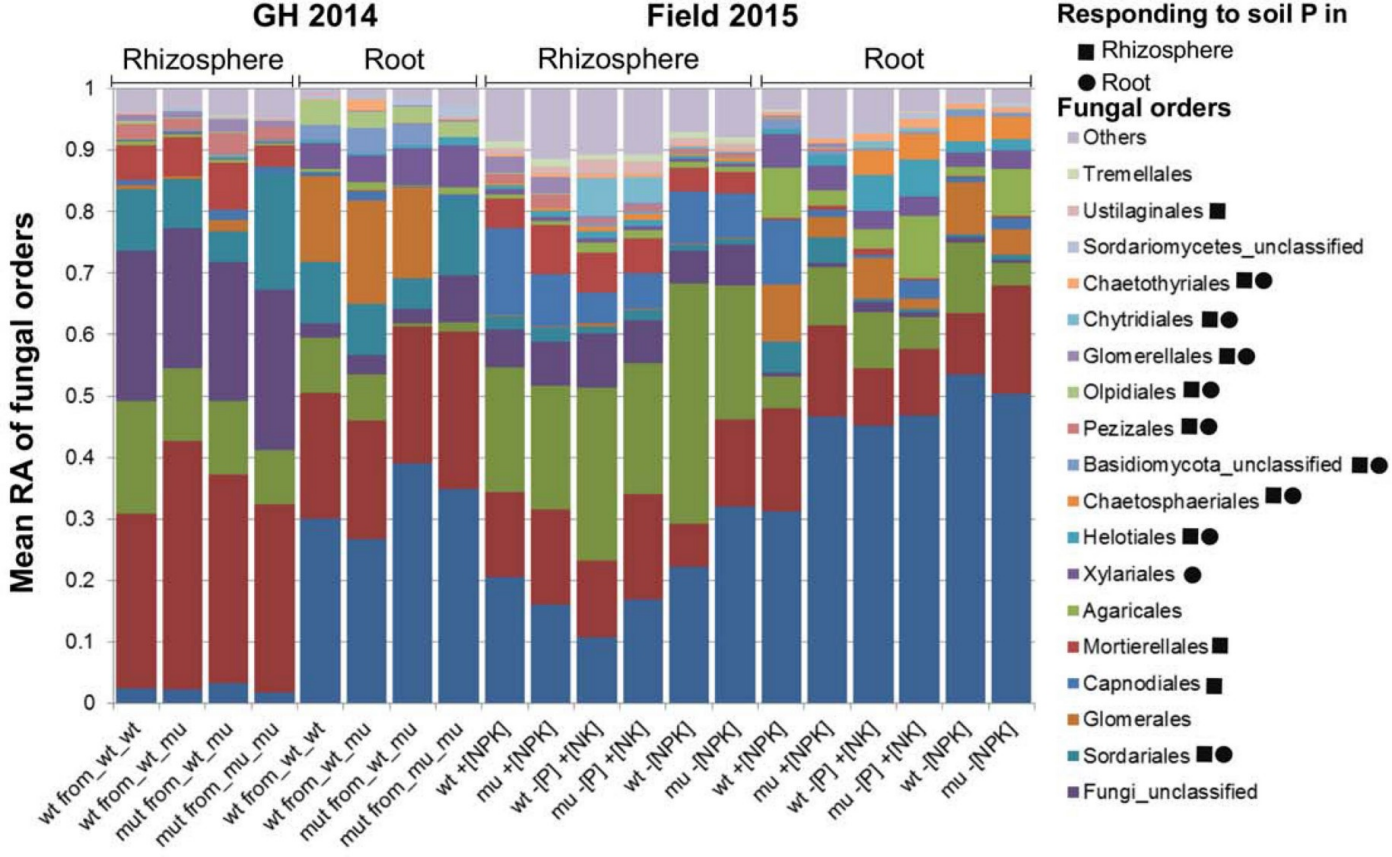
Relative abundance (percentage of total ITS2 reads) of fungal orders in rhizosphere and root samples in GH 2014 and Field 2015 experiments. The orders which were significantly affected by soil type in the Field 2015 in root or rhizosphere compartments are indicated with symbols (ANOVA *P* < 0.05)

**Fig 5:**
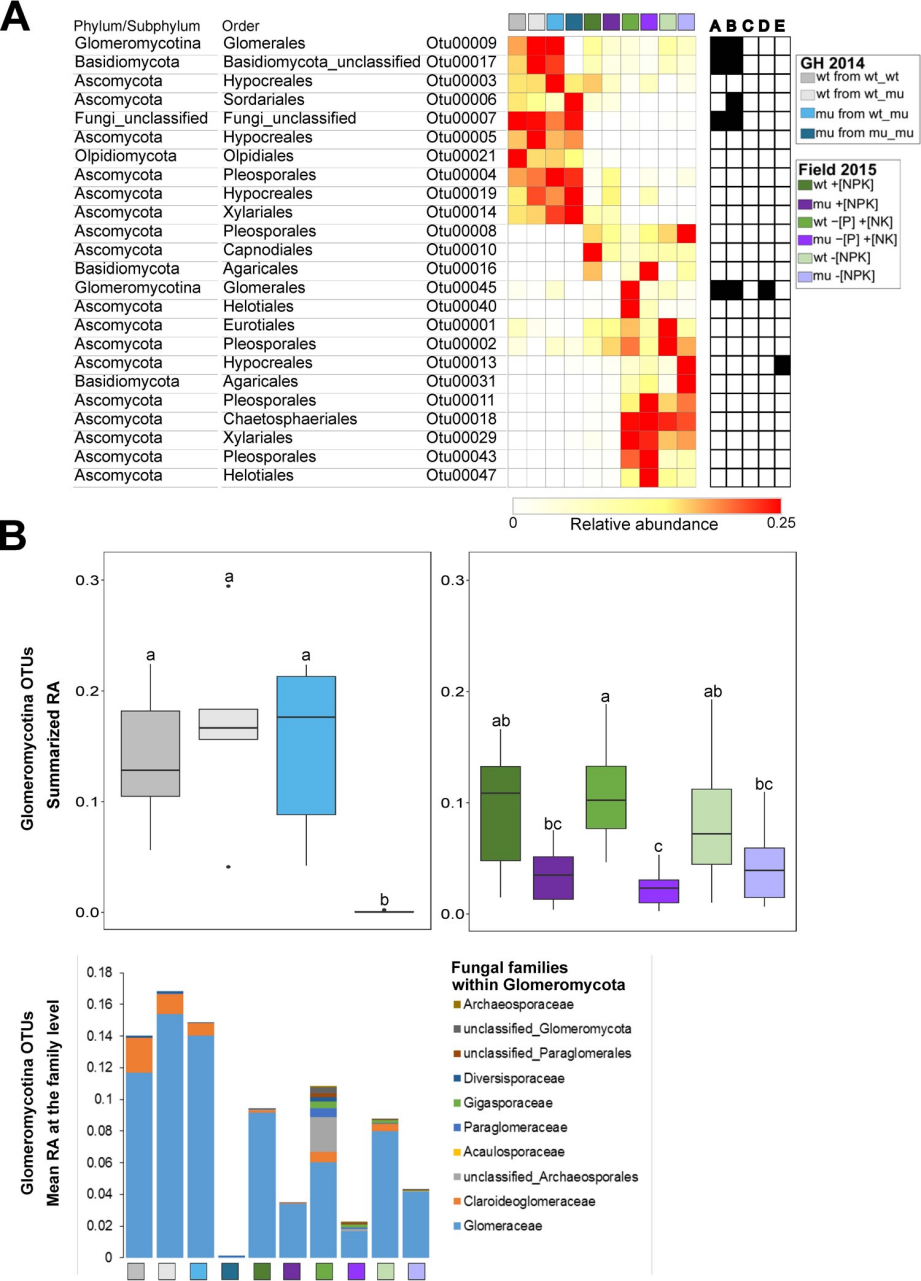
Average relative abundance (RA) of fungal OTUs. A) RA of fungal OTUs differing between wt and mutant roots identified by SIMPER analysis. The stripes on the right side of the plot indicate the P-value of binary comparisons (Wilcoxon test, black indicates significant *P* < 0.05, white non-significant *P*-value). Different letters indicate the comparisons performed: A: wt from wt_wt pot vs. mutant from mu_mu pot in GH 2014, B: mutant from mu_mu pot vs. mu from mu_wt pot in GH 2014, C: mutant vs. wt in +[NPK], D: mutant vs. wt in–P +[NK], E: mutant vs wt in–[NPK] field. B) Summarized RA of Glomeromycota OTUs and the community structure at the family level of Glomeromycota fungi in in root samples. Different letters indicate significant differences between the treatments (ANOVA followed by Tukey’s HSD test, *P* < 0.05).

We further analyzed the beta diversity patterns of fungal communities in each experiment separately. In the greenhouse experiment fungal community structure was affected mainly by the compartment type and by the plant configuration in the pots (i.e. wt_wt, wt_mu, mu_mu) (Fig 3 and S5 Table). The effect of the pot design was more pronounced within the root fungal communities than within the rhizosphere communities (PERMANOVA 21% and 13% of variance, respectively) (S5 Table). Examination of the fungal community structure at the order level revealed only minor differences between mu and wt plants grown in pots with a single genotype (i.e. wt_wt and mu_mu), whereas root fungal community profiles of mu plants grown with wt (wt_mu) resembled those of wt plants (Fig 4). The roots of mu plants from mu_mu pots were depleted in fungi belonging to the orders Glomerales, Paraglomerales and some unclassified Basidiomycota, whereas they were enriched in Savoryellales, Sordariomycetes and unclassified fungi compared to wt plants grown in monoculture (Fig 4). Considering the significant effect of genotype configuration in the pots on fungal community structure (S5 Table) and the differences observed at the order levels (Fig 4), we employed SIMPER analysis to depict the fungal OTUs which potentially differentially colonized wt and mu plants grown in uniform configuration (mu_mu or wt_wt). Here the difference between wt and mu was limited to two fungal OTUs colonizing roots belonging to the sub-phylum Glomeromycotina (OTU00045 and OTU00009), one to unclassified Basidiomycota OTU (OTU00017) with all three being more abundant in wt relative to mu, and one unclassified fungal OTU which was more abundant in mu roots compared to wt (Fig 5A; SIMPER with Wilcoxon test, *P* < 0.05). This shows that a defective MPU affected only a subset of root colonizing fungi, mostly limited to fungi belonging to the Glomeromycotina. This observation could be further confirmed by plotting the summarized relative abundance of OTUs belonging to Glomeromycotina (Fig 5B) which was lower in mutant roots compared to wt, corroborating our results on the differences in AM fungal colonization in mu and wt roots obtained upon staining of fungal structures (Fig 2). Importantly, the differences in fungal OTU abundances, the summarized average abundances of Glomeromycotina OTUs as well as the proportion of fungal families within Glomeromycotina in roots were equal in both genotypes when wt and mu plants were grown in the same pot (i.e. wt_mu; Fig 5A and 5B), hinting to the capacity of wt plants to serve as nurse plants in the pot system.

In the Field 2015 experiment the fungal community structure was affected primarily by the compartment type and by soil nutrient management, which could be observed in the PCoA on Bray–Curtis dissimilarities (Fig 3 and S6 Table). Soil nutrient management effects on fungal community structure were more pronounced in the rhizosphere relative to the root (S6 Table), suggesting a higher stability of fungal communities in roots compared to the soil-associated rhizosphere compartment in response to soil P content. Fungal orders the relative abundance of which was affected by soil nutrient management (ANOVA, *P* < 0.05, Fig 4) included the Sordariales, Capnodiales, Mortierellales, Chaetosphaeriales, Pezizales, Olpidiales and Glomerellales which were enriched in maize roots grown in +[NPK] soil compared to–[P] +[NK] or–[NPK] soils, and the Helotiales, Chaetosphaeriales, Chytridiales, Chaetothyriales and Ustilaginales which were enriched in maize roots grown in −[P] +[NK] or −[NPK] soil relative to +[NPK], suggesting that these fungal groups´ capacity to colonize maize roots depended on the soil P status.

The overall maize genotype effect that explained the variance in the microbiome in the Field 2015 experiment was minor but significant (PERMANOVA, 2% of variance), similarly to the interaction between genotype and soil (PERMANOVA, 2% of variance, S6 Table). However, within each soil nutrient management system, genotype effects were not evident, neither in the root nor in the rhizosphere compartments, suggesting that in mixed maize stands in the field, wt and mu plants assembled similar fungal communities (S6 Table). This was further corroborated at the fungal order level (Fig 4) with the exception of the Glomerales which were less abundant in mu relative to wt plants in plants grown in +[NPK] soil, while in −[P] +[NK] soil mu roots were depleted in Paraglomerales, Diversisporales, and unclassified fungi compared to wt (Fig 4). Similar trends were observed at the fungal family level within the Glomeromycotina subphylum, as in +[NPK] soil Glomeraceae were more abundant in the wt compared to mu roots, whereas in–[P] +[NK] soil fungi belonging to the families Paraglomeraceae, Gigasporaceae, Archaeosporaceae, Claroideoglomeraceae, and Diversisporaceae were more abundant in wt relative to mu roots (Fig 5B). In -[NPK] soil only the fungal OTUs belonging to Claroideoglomeraceae family were more abundant in wt than in mu roots (Fig 5B). These observations suggested that the impairment of MPU affected host interactions with fungal groups from the Glomeromycotina depending on the soil nutrient management. This analysis in combination with highest alpha diversity of Glomeromycotina fungi in wt roots in −[P] +[NK] soil revealed some degree of host preference within the Glomeromycotina. SIMPER analysis largely failed to recapitulate the differences in beta diversity captured at the order and family levels at the level of fungal species. OTU00045 (belonging to Glomerales) in −[P] +[NK] soil was more abundant in wt roots compared with mu while OTU00013 (Hypocreales) in -[NPK] soil was more abundant in mu compared with wt (Wilcoxon *P* < 0.05, FDR corrected, Fig 5A). The overall summarized abundance of Glomeromycotina OTUs was significantly lower in mu roots compared to wt in −[P] +[NK]soil (Fig 5B) and to a lower degree in +[NPK] and -[NPK] soils. Eventually we used the variation in leaf ionome in both experiments as a basis in an attempt to search for relationships between fungal groups and leaf nutritional status. A correlation analysis between elemental content in the leaf and fungal order abundances in the root revealed positive correlations between Fe/Ni and OTUs belonging to the Boliniales in the Field 2015 experiment and Ca/As/Mo and unclassified fungi/Saccharomycetales/Myrmecridiales OTUs in the GH 2014 experiment, whereas negative correlations were observed between Mn and Glomerellales, and Ca and Paraglomerales in the Field 2015 and GH 2014 experiment, respectively (S7 Table). Overall our results showed that a disruption of MPU in maize with its physiological consequences hardly affected the entire fungal community under field conditions. Changes in Glomeromycotina OTU abundances alluded to a particular role of the MPU in maintaining a growth promoting mycorrhizal microbiome with well-balanced taxonomic composition of AM fungi.

## Discussion

This work provides a comparison of mu and wt plants which were cultivated in non-sterile field soil that was inhabited by natural microbial communities. Comparative analysis of mu and wt allowed studying how maize plants deviating in MPU capacity integrated the information on nutrient and neighbor distributions into root-foraging for heterogeneously distributed nutrients, and how this affected nutrient acquisition, plant growth and interactions with soil microbiota. Taken together, the results showed that a disruption of the MPU pathway in maize resulted in a major impairment of plant growth, a differential allocation of nutrient elements to source leaves, and a reduction of AM fungal abundance in an otherwise robust root fungal microbiome exhibiting surprisingly little variation in fungal assemblages beyond AM fungi. The MPU pathway was previously shown to significantly contribute to total P uptake in mycorrhizal plants [17]. Relevant experiments were mainly performed in simplified systems in which single plants were grown in pots filled with sterilized soil with addition of AM inoculum often consisting of one fungal species [16,20,26]. Reverse genetics to determine the role of the *Pht1;6* gene showed a marked contribution of the MPU pathway to maize performance under field conditions [26]. This stands in accordance with the here presented results.

Photosynthetic carbon fixation takes place in the source leaves enabling the synthesis of primary metabolites, their allocation to sink tissues, biomass production and root exudation during vegetative growth [59]. In all three managed soils in the field and in plant pots wt growth performance exceeded that of mu in the mixed (wt-mu) configuration. Disruption of the mycorrhizal Pi uptake via Pht1;6 resulted in a more than two-fold reduction of P content under greenhouse and field conditions (Fig 1B) corroborating previous results [26,60].

Differences in the soil nutrient management translated into differential growth performance of wt and mu whereas P content in corresponding source leaves remained remarkably robust (Fig 1) which demonstrated the maintenance of P homeostasis in the source leaves across the managed soils. Similarly, the plant genotype configuration in plant pots hardly affected P content in the source leaf. It can´t be excluded that P taken up into the plant was translocated predominantly to sink tissues like proliferating roots and young sink leaves [26,60,61], which would explain why maize shoot growth does not necessarily correlate with P content in the first source leaf.

Wt plants which were grown in mixed culture in plant pots were significantly bigger than wt plants grown in monoculture, while both plants exhibited similar P content in their source leaves (Fig 1), suggesting that wt used the common mycorrhizal network (CMN), established by the AM fungi, highly efficiently for P uptake, at the expense of mu [62], and thus wt acted as the stronger sink for fungal P relative to mu. When grown in monoculture the two plants exploited the CMN on equal terms. With respect to the reciprocal transfer of C and P in the AM symbiosis it would be interesting to reveal the relative contribution of host genotype and mycobiont to nutrient fluxes within a maize stand. Our results of the plant pot experiment suggested that neighboring maize plants strongly compete for mycorrhizal P and that an operating MPU provides fitness benefits to the mycorrhizal host in low P soil substrate.

Mutant plants exhibited imbalances in nutrient elemental contents compared to wt especially under field conditions (S2 Fig). Differences in elemental fingerprints were observed previously in maize plants growing on the same fields [26], and together with the here presented results the relevance of MPU for differential acquisition of mineral elements and their subsequent allocation to the shoot was demonstrated under field conditions. The profiles of shoot P, S and Cu contents across the two genotypes and soil managements were inversely correlated with those of K, Mn and Fe especially at low P conditions. We could thus conclude that acquisition/uptake strategies for elements sharing similar elemental profile patterns were differentially affected by MPU.

MPU activity affected not only plant growth and leaf elemental content, but also root colonization with AM fungi (Fig 2). In the pot the inability of arbuscules to form properly in mu monoculture could be rescued by the presence of wt [26]. This transcomplementation (or ‘nurse plant’) effect was likely due to the transfer of C from wt to hyphae colonizing mu via the CMN, driven by active MPU in wt plants in accordance with the theory of reciprocal rewards in AM symbiosis [33,63]. This was corroborated by previous results with *pht1;6* mutants grown in mixed culture with mycorrhizal chive plants in pots inoculated with the AM fungal species *Rhizophagus irregluaris* [26]. In that experimental set up, the use of radioactive tracer P demonstrated that the arbuscules formed in mu roots were non-functional [26]. An obvious explanation for the absence of trans-complementation in the field setting (Fig 2) could be the absence of nurse effects of wt plants, i.e. provision of photosynthetic carbon to fungal colonizers which simultaneously colonized mu roots, due to the planting distance which prevented overlapping of large portions of mu and wt mycorrhizospheres in the mixed stands. We concluded that in the presence of natural microbiota mycorrhizal host plants equipped with an efficient MPU support AM fungal colonization and arbuscule formation in less-efficient neighboring plants through the delivery of C and energy to the CMN also when arbuscules in neighboring plants are dysfunctional.

Cross-validation of the Illumina sequencing data obtained on the same DNA samples revealed close agreement between ARISA and Illumina sequencing output, especially in their ability to discriminate samples from different root compartments. We thus concluded that ARISA can be used as an inexpensive and rapid way for the analysis of fungal diversity and community fingerprinting in rhizosphere and root endosphere samples, and to study interdependencies between microbial community structure and applied experimental conditions prior to performing amplicon sequencing [64,65]. However, ARISA seemed to underestimate sample richness. In general, small differences in fungal species´ abundance between mutant and wt plants (Fig 5A) could be predicted already by ARISA, although the average number of fungal phylotypes (potential species) was lower in ARISA (141) compared to the number of sequenced OTUs (256, S3 Table). We could thus show that fungal community profiling by ARISA can allow selection of suitable experimental treatments and reduce sample costs before final OTU sequencing.

Furthermore, we observed that fungal communities in root and rhizosphere were affected by the soil P fertilization management in the field (Figs 3 and 4 and S6 Table). Fungi belonging to the Helotiales order were more abundant in roots of maize grown in −[P] +[NK]) or −[NPK] soil compared to +[NPK], whereas the opposite trend was apparent for Olpidiales fungi. Similar observations were made in Brassicaceae plants grown in short-term fertilized NK soil [51]. Helotiales fungi have high agroecological potential because they were shown to mediate P transfer to the plant host and promote plant growth [48]. The NPK fertilizer management did not affect the overall abundance of Glomeromycotina OTUs in roots, but it affected the Glomeromycotina community composition (Fig 5B). There are some contrasting reports on Glomeromycotina fungi behavior in maize roots in response to soil P fertilization. For instance Gomes et al. (2018) observed an increased abundance of Glomeromycotina in maize roots in response to low P fertilization [66], whereas Yu et al. (2018) reported an increase of Glomeromycotina abundance in maize roots under high P fertilization [41]. Our results implicated MPU activity and the host nutrient status [51] in the control of AM fungal colonization of maize roots in response to P fertilization.

The difference in fungal community structure in *pht1;6* vs. wt plants was most apparent in the GH 2014 experiment with maize grown in monoculture (Fig 5A). Here two Glomeromycotina OTUs (OTU00045 and OTU00009) and one Basidiomycota OTU were depleted in mutant roots, whereas unclassified fungal OTUs were enriched. This difference in relative fungal abundances in wt and mu was not apparent in plants grown in the field in 2015 (Fig 5A), which could be explained by overall higher nutrient content in source leaves in the field and a reduced nutrient status in plants grown in pots (Fig 1 and S2 Fig). Previously, a statistically significant small effect of the development of functional arbuscules on root fungal communities was observed in the legume *Lotus japonicus*, in which depletion of Glomeromycotina OTUs in roots resulted in increased abundance of Ascomycota fungal species e.g. belonging to the orders Hypocreales and Helotiales [42,43]. In these studies mutants affected in genes acting upstream of mycorrhiza-dependent Pi transport were explored. The currently still limited number of studies allow to suggest that the functionality of arbuscules and the activity of mycorrhiza-specific Pi transporters play a major role in shaping the community of AM fungi in roots while they leave non-AM mycobiota largely unaffected. Similarly, in field-grown tobacco plants in which *CCaMK* of the CSSP was silenced, the fungal and bacterial communities in roots were similar to the wt plants [45]. It is possible that we failed to detect small effects of MPU on root fungal communities in the field experiment because of variance introduced by uncontrolled environmental conditions and stochastic effects. Increasing the number of experiments and the number of replicates could provide sufficient power to identify the specific taxa that respond to differences in maize genotypes [37,38]. Nevertheless, our results show that the non-AM fungal mycobiome is robust and only weakly affected by AM symbiosis.

This work provided insight into the mechanistic basis of variation in symbiotic (microbiomic) outcome and the importance of MPU for agricultural productivity in low P managed agronomic systems. Moreover, it revealed the potential of optimizing P uptake for crop improvement in cereals with MPU as a central target of selection. Based on correlation between fungal order abundances and leaf ionomic content it was interesting to find a few fungal groups in the root which may have been involved in the acquisition of specific elements in leaves or *vice versa* which may have responded to changes in the leaf ionome. For example, Boliniales fungi correlated with Fe/Ni content and Glomerellales OTUs with Mn content. The Boliniales are an order of fungi within the class Sordariomycetes (Ascomycota) to which also the beneficial fungus *Colletotrichum tofieldiae* belongs to and which was shown to transport P to host roots [67]. Glomerellales species were shown to oxidize Mn(II) which can affect Mn biomineralization [68]. However, as correlation does not imply causation more work is needed for a detailed validation of these correlations including microbiomics and reductionist approaches with fungal isolates belonging to these orders to understand the complex interplay between microbes, hosts, and biogeochemistry.

Based on our results, we propose the testable hypothesis that the enhancement of mycorrhizal Pi uptake has a pronounced effect on plant biomass production with small impact on root microbial diversity in soils characterized by low input of P fertilizer, thus allowing an increase in yields without pronounced ecosystem changes. In sum our comparative study revealed that competition for resources between neighboring mycorrhizal plants can be more pronounced in pots than in the field which points to the importance of combined approaches with greenhouse and field experiments in research investigating the interplay between plant nutrient uptake, growth and root microbiota. Finally, this work provided some evidence that the holobiont concept must take neighboring plants and the striking role of obligate biotrophic mutualistic fungi in effects on plant growth and on the process of microbiota assembly into account to be complete.

## Supporting information

S1 Fig Plant growth in GH 2014 and Field 2015 experiments. A) Experimental setup of the greenhouse experiment 2014 (GH 2014). The circle represents the pot, the genotypes are color-coded. B) Experimental setup of field 2015 experiment. The square represents a single maize plant, the genotypes are color-coded, the soil in which the plants were grown is indicated on the left side of the graph. The number of samples collected in each experiment is indicated in S1 and S2C Tables) Exemplary pictures of pots in GH 2014 experiment at the day of sampling. D) Exemplary pictures of plants grown under different fertilizer management practices in the Field 2015 experiment.
(EPS)

S2 Fig Concentration of elements in source leaves of wt and *pht1;6* plants. Only ions for which significant differences were found are shown. Different letters indicate significant differences between the treatments (ANOVA followed by Tukey’s HSD test, *P* < 0.05, n = 5 in GH 2014 or n = 8–10 in Field 2015 experiment).
(EPS)

S3 Fig ARISA-based comparison of microbial communities colonizing roots of *pht1;6* mutant and wt roots in +[NPK] soil in GH 2014 experiment visualized using principal coordinates analysis (PCoA) on Bray-Curtis dissimilarities. (EPS)

S4 Fig Alpha diversity (Shannon index) in root and rhizosphere samples in GH 2014 and Field 2015 experiments. Different letters indicate significant differences between the treatments (ANOVA followed by Tukey’s HSD test, *P* < 0.05).
(EPS)

S5 Fig Principal coordinates analysis (PCoA) on Bray-Curtis dissimilarities of fungal communities in root and rhizosphere samples in GH 2014 and Field 2015 experiments. (EPS)

S1 Table Pot number in GH 2014 experiment. (DOCX)

S2 Table Number of root and rhizosphere samples collected in the Field 2015 experiment and used for fungal microbiota analysis. (DOCX)

S3 Table Sequencing analysis summary. (DOCX)

S4 Table PERMANOVA on Bray-Curtis dissimilarities of fungal community structure in GH 2014 experiment and in Field 2015 experiment. (DOCX)

S5 Table PERMANOVA on Bray-Curtis dissimilarities of root-associated fungal community in greenhouse experiment 2014 (GH2014). (DOCX)

S6 Table PERMANOVA on Bray-Curtis dissimilarities of root-associated fungal community in field experiment 2015. (DOCX)

S7 Table Person’s correlation between leaf nutrient content and fungal orders abundance in roots. (DOCX)

S8 Table Concentration of elements in source leaves of wt and mu plants in mg/kg dry weight. n.a.indicates not applicable, n.m.—not measured.
(XLSX)
