# Supplementary material for: Neighboring plants divergently modulate effects of loss-of-function in maize mycorrhizal phosphate uptake on host physiology and root fungal microbiota
Source: PLoS One. 2020 Jun 17;15(6):e0232633. doi: 10.1371/journal.pone.0232633 (PMC7299352; doi:10.1371/journal.pone.0232633)
Supplement: S1 Table — (DOCX) [file pone.0232633.s006.docx]

Table S1. Pot number in GH 2014 experiment. Two plants were grown in one pot, corresponding genotypes are indicated in the column heading (wt_wt; two B73 plants grown in one pot; mu_mu; two *pht1;6* mutants grown in one pot, wt_mu; B73 and *pht1;6* grown in one pot). Each plant from one pot was sampled separately, followed by root and rhizosphere separation and used for fungal microbiota analysis.

| **Compartment** | **Pot design** | | |
| --- | --- | --- | --- |
|  | **wt_wt** | **mu_mu** | **wt_mu** |
| root | 5 | 5 | 5 |
| rhizosphere | 5 | 5 | 5 |
