# Supplementary material for: Neighboring plants divergently modulate effects of loss-of-function in maize mycorrhizal phosphate uptake on host physiology and root fungal microbiota
Source: PLoS One. 2020 Jun 17;15(6):e0232633. doi: 10.1371/journal.pone.0232633 (PMC7299352; doi:10.1371/journal.pone.0232633)
Supplement: S2 Table — (DOCX) [file pone.0232633.s007.docx]

Table S2. Number of root and rhizosphere samples collected in the Field 2015 experiment and used for fungal microbiota analysis.

|  | **Soil nutrient management** | | |
| --- | --- | --- | --- |
| **Genotype/compartment** | **-[NPK]** | **-[P] +[NK]** | **+[NPK]** |
| wt |  |  |  |
| root | 10 | 10 | 8 |
| rhizosphere | 10 | 10 | 10 |
|  |  |  |  |
| mu |  |  |  |
| root | 9 | 10 | 8 |
| rhizosphere | 9 | 10 | 9 |
