# Supplementary material for: Neighboring plants divergently modulate effects of loss-of-function in maize mycorrhizal phosphate uptake on host physiology and root fungal microbiota
Source: PLoS One. 2020 Jun 17;15(6):e0232633. doi: 10.1371/journal.pone.0232633 (PMC7299352; doi:10.1371/journal.pone.0232633)
Supplement: S3 Table — (DOCX) [file pone.0232633.s008.docx]

Table S3. Sequencing analysis summary.

| **Sample** | **Total reads ^a^** | | **Fungal reads ^b^** | | **Fungal OTUs ^c^** | |
| --- | --- | --- | --- | --- | --- | --- |
|  | **reads** | **St. dev.** | **reads** | **St. dev.** | **OTU number** | **St. dev.** |
| rhizosphere | 40149 | 14799 | 36528 | 13336 | 602 | 120 |
| root | 41909 | 15921 | 41344 | 15603 | 256 | 78 |

a Number of reads per sample after chimera and low abundant OTU removal.

b Final number of fungal reads per samples after removal of non-fungal reads.

c Fungal OTU number per sample after removal of non-fungal reads.
