# Supplementary material for: Neighboring plants divergently modulate effects of loss-of-function in maize mycorrhizal phosphate uptake on host physiology and root fungal microbiota
Source: PLoS One. 2020 Jun 17;15(6):e0232633. doi: 10.1371/journal.pone.0232633 (PMC7299352; doi:10.1371/journal.pone.0232633)
Supplement: S4 Table — (DOCX) [file pone.0232633.s009.docx]

Table S4. PERMANOVA on Bray-Curtis dissimilarities of fungal community structure in GH 2014 experiment and in Field 2015 experiment. PERMANOVA model used: compartment x experiment /soil nutrient management x genotype.

| **Factor** | **Variance explained** | ***P*-value** |
| --- | --- | --- |
| **Overall** |  |  |
| compartment (root, rhizosphere) | 27% | 1 x 10^-5^ |
| Experiment (GH2014, Field2015) | 21% | 1 x 10^-5^ |
| genotype (wt, mu) | 1% | 1 x 10^-5^ |
| compartment x experiment | 8% | 1 x 10^-5^ |
| compartment x genotype | 0.6% | 8 x 10^-3^ |
| compartment x experiment x soil | 9% | 1 x 10^-5^ |
| compartment x experiment x genotype |  | 0.52 |
| compartment x experiment x soil x genotype | 2% | 0.003 |
|  |  |  |
| **Root** |  |  |
| experiment | 32% | 1 x 10^-5^ |
| genotype | 3% | 7 x 10^-4^ |
| experiment x soil | 13% | 1 x 10^-5^ |
| experiment x genotype |  | 0.13 |
|  | 3% | 0.01 |
|  |  |  |
| **Rhizosphere** |  |  |
| experiment | 48% | 1 x 10^-5^ |
| genotype | 2% | 0.01 |
| experiment x soil | 12% | 1 x 10^-5^ |
| experiment x genotype |  | 0.36 |
| experiment x soil x genotype |  | 0.17 |
