# Supplementary material for: Neighboring plants divergently modulate effects of loss-of-function in maize mycorrhizal phosphate uptake on host physiology and root fungal microbiota
Source: PLoS One. 2020 Jun 17;15(6):e0232633. doi: 10.1371/journal.pone.0232633 (PMC7299352; doi:10.1371/journal.pone.0232633)
Supplement: S5 Table — (DOCX) [file pone.0232633.s010.docx]

Table S5. PERMANOVA on Bray-Curtis dissimilarities of root-associated fungal community in greenhouse experiment 2014 (GH2014). PERMANOVA model used: compartment x pot / genotype.

| **Factor** | **Variance explained** | ***P*-value** |
| --- | --- | --- |
| **Overall** |  |  |
| compartment (root or rhizosphere) | 54% | 1 x 10^-5^ |
| pot design (wt_wt, wt_mu, mu_mu) | 6% | 0.003 |
| compartment x pot design |  | 0.17 |
| compartment x pot design x genotype |  | 0.31 |
|  |  |  |
| **Root** |  |  |
| pot design | 21% | 1 x 10^-5^ |
| pot design x genotype |  | 0.22 |
|  |  |  |
| **Rhizosphere** |  |  |
| pot design | 13% | 0.03 |
| pot design x genotype |  | 0.44 |
