# Supplementary material for: Neighboring plants divergently modulate effects of loss-of-function in maize mycorrhizal phosphate uptake on host physiology and root fungal microbiota
Source: PLoS One. 2020 Jun 17;15(6):e0232633. doi: 10.1371/journal.pone.0232633 (PMC7299352; doi:10.1371/journal.pone.0232633)
Supplement: S6 Table — (DOCX) [file pone.0232633.s011.docx]

Table S6. PERMANOVA on Bray-Curtis dissimilarities of root-associated fungal community in field experiment 2015. PERMANOVA model used: compartment x soil nutrient management x genotype.

| **Factor** | **Variance explained** | ***P*-value** |
| --- | --- | --- |
| **Overall** |  |  |
| compartment (root or rhizosphere) | 41% | 1 x 10^-5^ |
| soil (-[NPK], -[P] +[NK], +[NPK]) | 11% | 1 x 10^-5^ |
| genotype | 2% | 0.003 |
| compartment x soil | 6% | 1 x 10^-5^ |
| compartment x genotype |  | 0.08 |
| soil x genotype | 2% | 0.005 |
| compartment x soil x genotype |  | 0.33 |
|  |  |  |
| **Root** |  |  |
| soil | 25% | 1 x 10^-5^ |
| genotype | 4% | 7 x 10^-4^ |
| soil x genotype | 6% | 0.001 |
|  |  |  |
| **Rhizosphere** |  |  |
| soil | 31% | 1 x 10^-5^ |
| genotype | 4% | 0.006 |
| soil x genotype |  | 0.12 |
