# Supplementary material for: Neighboring plants divergently modulate effects of loss-of-function in maize mycorrhizal phosphate uptake on host physiology and root fungal microbiota
Source: PLoS One. 2020 Jun 17;15(6):e0232633. doi: 10.1371/journal.pone.0232633 (PMC7299352; doi:10.1371/journal.pone.0232633)
Supplement: S7 Table — (DOCX) [file pone.0232633.s012.docx]

Table S7. Person’s correlation between leaf nutrient content and fungal orders abundance in roots. Only significant correlations were considered (*P* < 0.05, FDR corrected, Pearson’s rho > 0.6)

| **Experiment** | **Variable 1** | **Variable 2** | **Pearson’s rho** | ***P*-value** |
| --- | --- | --- | --- | --- |
| **GH 2014** |  |  |  |  |
|  | Mo | Myrmecridiales | 0.66 | 0.00014 |
|  | As | Saccharomycetales | 0.63 | 0.00038 |
|  | Ca | Fungi_unclassified | 0.62 | 0.00057 |
|  | Ca | Paraglomerales | -0.60 | 0.00079 |
| **Field 2015** |  |  |  |  |
|  | Fe | Boliniales | 0.65 | 1.29E-07 |
|  | Ni | Boliniales | 0.63 | 5.12E-07 |
|  | Mn | Glomerellales | -0.61 | 1.56E-06 |
